# Clinical features and prognostic factors in patients diagnosed with lymphovascular invasion of testicular germ-cell tumors: Analysis based on the SEER database

**DOI:** 10.3389/fonc.2023.1142441

**Published:** 2023-03-03

**Authors:** Hu Ke, Shengming Jiang, Ziqi He, Qianlin Song, Dashuai Yang, Chao Song, Caitao Dong, Junwei Liu, Xiaozhe Su, Jiawei Zhou, Yunhe Xiong

**Affiliations:** ^1^ Urology Department, Renmin Hospital of Wuhan University, Wuhan, Hubei, China; ^2^ Department of Hepatobiliary Surgery, Renmin Hospital of Wuhan University, Wuhan, China

**Keywords:** testicular germ cell tumor, lympho-vascular invasion, SEER, overall survival, nomogram, prognosis

## Abstract

**Background:**

Lymphovascular invasion (LVI) is a high-risk factor for testicular germ-cell tumors (TGCT), but a prognostic model for TGCT-LVI patients is lacking. This study aimed to develop a nomogram for predicting the overall survival (OS) of TGCT-LVI patients.

**Methods:**

A complete cohort of 3288 eligible TGCG-LVI patients (training cohort, 2300 cases; validation cohort, 988 cases) were obtained from the Surveillance, Epidemiology, and End Results database. Variables screened by multivariate Cox regression analysis were used to construct a nomogram, which was subsequently evaluated using the consistency index (C-index), time-dependent receiver operating characteristic curve (ROC), and calibration plots. The advantages and disadvantages of the American Joint Committee on Cancer (AJCC) staging system and the nomogram were assessed by integrated discrimination improvement (IDI) and net reclassification improvement (NRI). Decision-analysis curve (DCA) was used to measure the net clinical benefit of the nomogram versus the AJCC staging system. Finally, Kaplan–Meier curves were used to evaluate the ability to identify different risk groups between the traditional AJCC staging system and the new risk-stratification system built on the nomogram.

**Results:**

Nine variables were screened by multivariate Cox regression analysis to construct the nomogram. The C-index (training cohort, 0.821; validation cohort, 0.819) and time-dependent ROC of 3-, 5-, and 9-year OS between the two cohorts suggested that the nomogram had good discriminatory ability. Calibration curves showed good consistency of the nomogram. The NRI values of 3-, 5-, and 9-year OS were 0.308, 0.274, and 0.295, respectively, and the corresponding values for the validation cohort were 0.093, 0.093, and 0.099, respectively (P<0.01). Additionally, the nomogram had more net clinical benefit as shown by the DCA curves, and the new risk-stratification system provided better differentiation than the AJCC staging system.

**Conclusions:**

A prognostic nomogram and new risk-stratification system were developed and validated to assist clinicians in assessing TGCT-LVI patients.

## 1 Introduction

Testicular tumors are relatively uncommon, accounting for only 1% of all male tumors (1), but they are the most common solid tumors in men aged 20–34 years. In recent years, the global incidence of testicular tumors has steadily increased, especially in developed countries (2). Compared with only 1 per 100,000 men in Africa and Asia, the incidence rates in Norway, Denmark, and Switzerland were 9.9, 9.4, and 9.2 per 100,000 men, respectively (3). A total of 9910 new cases of testicular tumors were also diagnosed in the United States in 2022 (4). However, the reasons for this increase are not well documented (2, 5–8). The causes of testicular tumors remain unclear, and studies have identified cryptorchidism, hypospadias, low sperm count, and genetics as risk factors (9). The AJCC staging system is internationally used to assess the stage of testicular-tumor patients for subsequent treatment decision making and prognosis assessment.

Testicular germ-cell tumor (TGCT), comprising 95% of malignant tumors originating from the testicle, can be divided into seminoma tumors and non-seminoma tumors by histologic type (2, 10). The latter includes primarily embryonal carcinoma, choriocarcinoma, yolk sac tumor, teratoma, and four subtypes of mixed tumors, all of which have higher aggressiveness than the former (1). The main treatment method of TGCT is radical orchiectomy (11), and with corresponding adjuvant radiotherapy or chemotherapy, patients have a high cure rate (12, 13). However, based on the stratified assessment of tumor risk factors, lymphovascular invasion (LVI) is a high-risk factor for TGCT with occult metastases, thereby leading to higher recurrence rates and poorer prognosis. It is also important in assessing metastases and prognosis during the progression of the disease (14–16). Studies have shown that age, race, pathological type, and tumor size are associated with the recurrence and poor prognosis of testicular tumor. However, no articles describe the clinical characteristics and prognostic factors of patients with TGCT and LVI. Therefore, individualized predictive models are needed for TGCT patients with LVI.

Nomograms have been developed to predict the prognosis of cancer patients (17–19). Compared with the commonly used AJCC staging system, a nomogram incorporates more prognostic-related factors, which is more advantageous in terms of predictive accuracy and precision. The present study aimed to develop a nomogram to predict the prognosis of patients with TGCT-LVI and to explore its role in promoting personalized medicine and easy use by clinicians.

## 2 Patients and methods

### 2.1 Data sources and patient selection

**Figure 1:**
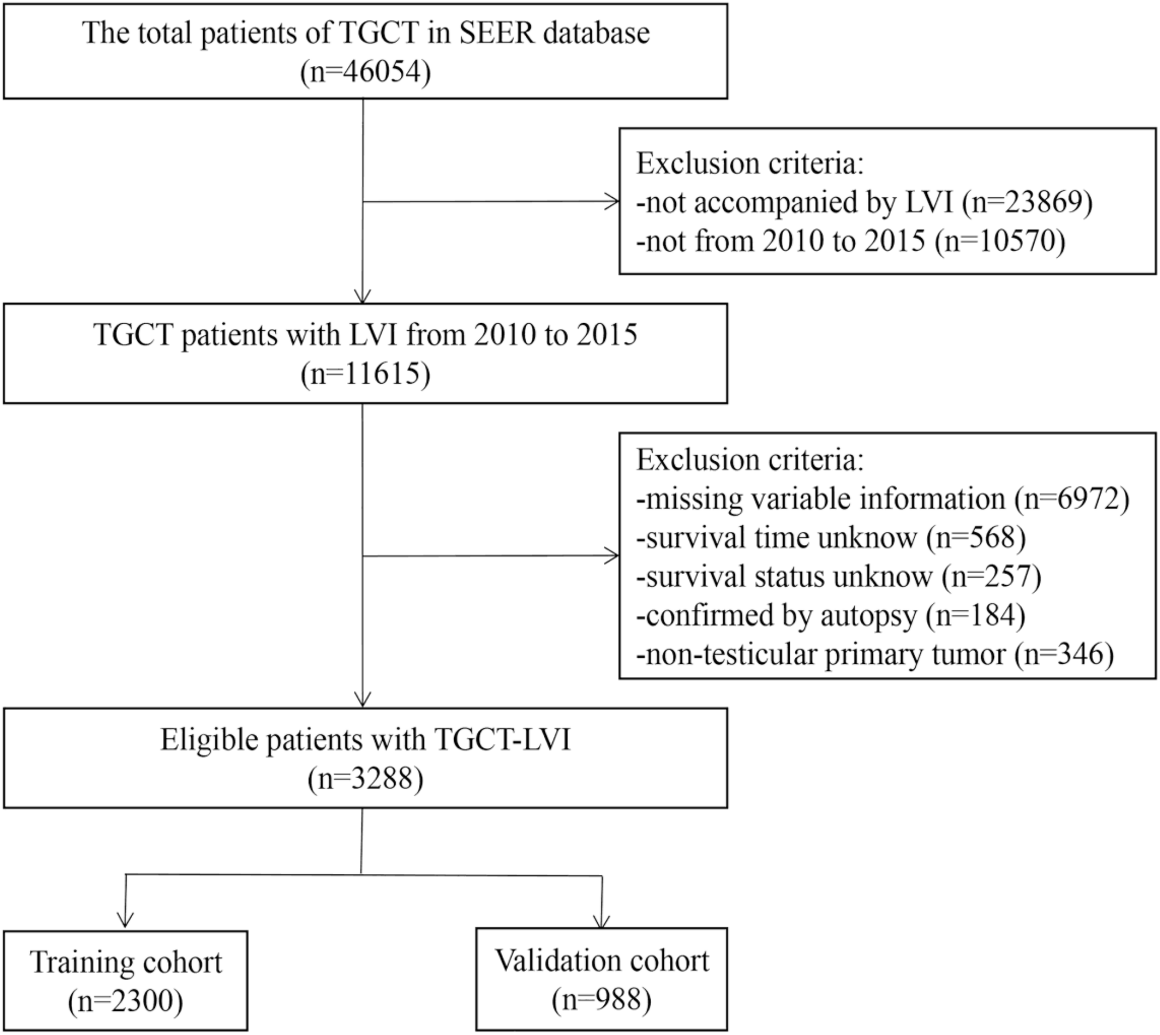
The screening process for the data of TGCT patients from the SEER database.

The Surveillance, Epidemiology, and End Results (SEER) database (https://seer.cancer.gov/) is a population-based database founded by the National Cancer Institute. The database is publicly available, so it does not need to pass ethical scrutiny when using data. SEER*Stat 8.4.0.1 software was applied to extract all information of TGCT patients from the dataset of incidence, i.e., SEER Research Plus Data 17 registries, Nov 2021 Sub (2000–2019). We selected patients diagnosed with TGCT-LVI between 2010 and 2015. The inclusion criteria were as follows: (i) ICD -O-3 morphology codes were 9060/3-9099/3; (ii) radical orchiectomy had been performed; (iii) postoperative pathological tissue was confirmed as malignant; (iv) the primary site is the testicles; and (v) complete follow-up information. The exclusion criteria were as follows: (i) missing variable information extracted; (ii) not accompanied by LVI; (iii) the time and status of survival are unknown; and (iv) confirmed only upon autopsy. The screening process for the data is shown in **Figure 1**.

### 2.2 Variable coding

The variables selected for this study were transformed into categorical variables. Age at diagnosis was stratified into four groups (<25, 25–44, 45–64, and >64 years). Race was separated as Black, White, and other. Marital status was divided into single, married, SDW (separated, divorced, or widowed), and unknown. Tumor primary site (undescended testis, descended testis and testis, NOS), laterality (left and right), histologic type (seminoma and non-seminoma), tumor size (0–4 cm, >4 cm), and tumor number (1 and >1). Radiotherapy, chemotherapy, and distant metastases (bone, brain, liver, and lung) were categorized according to whether to happen. This study used two tumor-grading methods, namely, Derived American Joint Committee on Cancer (AJCC) Stage Group, 7th ED (2010–2015) and Summary Stage 2000 (1988+). The Summary Stage, renamed by us as SEER stage, is used in the SEER cancer-statistics review and more recent SEER publications (including localized, regional, and distant. However, these indicators were not included in this study because of database limitations, missing information on lymph-node lesions and tumor surgical margins, and the TNM stage and serum tumor markers (AFP, HCG, and LDH) of testicular tumors being reflected by AJCC stage.

### 2.3 Cohort definition and model building

The R function “createDataPartition” was applied to randomly divide all patients into training and validation cohorts with a ratio of 7:3. The training cohort was used to screen the variables to construct the model, and the validation cohort was used to validate the results obtained by the training cohort. Univariate and multivariate Cox regression analyses were used to analyze the correlation between the variables screened in the training cohort and overall survival (OS) and subsequently calculate the risk ratios (HR) and the corresponding 95% confidence interval (CI). Then, the effective variables were screened out on the basis of P<0.05, and they were incorporated to construct a prognostic nomogram, which predicted the OS of TGCT-LVI patients at 3-, 5-, and 9-years.

### 2.4 Verification and calibration of nomograms

We used the consistency index (C-index) to evaluate the predictive ability of the nomogram. The receiver operating characteristic curve (ROC)–area under the curve (AUC) was used to assess the sensitivity and specificity of the model. The range of the C-index and AUC is 0.5–1, and >0.7 generally indicates a reasonable estimate. A calibration plot was used to visually compare the predicted prognosis of the nomogram with the actual prognosis. Decision-curve analysis (DCA) was used to measure the clinical application value of the nomogram, and the advantages and disadvantages of the nomogram and AJCC staging were compared using net reclassification improvement (NRI), integrated discrimination improvement (IDI), and C-index.

### 2.5 Statistical analysis

All variables in both cohorts were tested with the chi-square test. When the P-value was <0.05, the difference was statistically significant. The optimal cutoff for the total scores was determined with X-Tile (version 3.6.1). The relevant statistics packages were downloaded and used in an R (version 4.1.3) environment (https://www.r-project.org/).

## 3 Results

### 3.1 Baseline characteristics

Relevant clinical data of 46,054 TGCT patients were obtained from the SEER database. Based on the screening criteria described above, a complete analysis cohort of 3288 eligible TGCG-LVI patients (training cohort, 2300; validation cohort, 988) was included. **Table 1** shows the counts, proportions, and chi-square tests results of TGCT-LVI patients’ demographic characteristics in the training and validation groups. Among them, 3008 were white, 1732 were unmarried, and 1603 patients had testicles that descended into the scrotum normally. According to histologic type, seminoma and non-seminoma accounted for 43.1% and 56.9%, respectively. Among all patients undergoing radical orchiectomy, 358 cases received adjuvant radiotherapy, and 1845 received adjuvant chemotherapy. Patients with distant metastases at different sites accounted for <10%. Tumors >4 cm and single tumors were in the majority in both cohorts. Chi-square test results showed no difference in the distribution of baseline demographic and clinical features of TGCT-LVI patients in the two cohorts.

**Table 1:** Baseline characteristics for 3288 patients of testicular germ cell tumors with lymphovascular invasion (TGCT-LVI).

Characteristic	Whole population [n (%)]	Training cohort [n (%)]	Validation cohort [n (%)]	P value
Total	3288	2300	988	
Age(year)				
<25	757 (23%)	519 (22.6%)	238 (24.1%)	0.58
25-44	1937 (58.9%)	1355 (58.9%)	582 (58.9%)	
45-64	551 (16.8%)	397 (17.3%)	154 (15.6%)	
>64	43 (1.3%)	29 (1.3%)	14 (1.4%)	
Race				
Black	101 (3.1%)	68 (3%)	33 (3.3%)	0.81
White	3008 (91.5%)	2105 (91.5%)	903 (91.4%)	
Other	179 (5.4%)	127 (5.5%)	52 (5.3%)	
Marital status ^a^				
Single	1732 (52.7%)	1193 (51.9%)	539 (54.6%)	0.31
Married	1230 (37.4%)	866 (37.7%)	364 (36.8%)	
SDW	166 (5%)	124 (5.4%)	42 (4.3%)	
Unknown	160 (4.9%)	117 (5.1%)	43 (4.4%)	
Tumor primary site				
Undescended testis	46 (1.4%)	31 (1.3%)	15 (1.5%)	0.06
Descended testis	1603 (48.8%)	1090 (47.4%)	513 (51.9%)	
Testis, NOS ^b^	1639 (49.8%)	1179 (51.3%)	460 (46.6%)	
Laterality				
left	1559 (47.4%)	1073 (46.7%)	486 (49.2%)	0.18
Right	1729 (52.6%)	1227 (53.3%)	502 (50.8%)	
Histology				
Seminoma	1417 (43.1%)	1015 (44.1%)	402 (40.7%)	0.07
Nonseminoma	1871 (56.9%)	1285 (55.9%)	586 (59.3%)	
SEER stage				
Localized	1873 (57%)	1317 (57.3%)	556 (56.3%)	0.82
Regional	933 (28.4%)	651 (28.3%)	282 (28.5%)	
Distant	482 (14.7%)	332 (14.4%)	150 (15.2%)	
AJCC stage				
I	2146 (65.3%)	1501 (65.3%)	645 (65.3%)	0.25
II	602 (18.3%)	434 (18.9%)	168 (17%)	
III	540 (16.4%)	365 (15.9%)	175 (17.7%)	
Radiation				
No	2930 (89.1%)	2035 (88.5%)	895 (90.6%)	0.08
Yes	358 (10.9%)	265 (11.5%)	93 (9.4%)	
Chemotherapy				
No	1443 (43.9%)	1026 (44.6%)	417 (42.2%)	0.20
Yes	1845 (56.1%)	1274 (55.4%)	571 (57.8%)	
Metastasis of bone				
No	3266 (99.3%)	2286 (99.4%)	980 (99.2%)	0.52
Yes	22 (0.7%)	14 (0.6%)	8 (0.8%)	
Metastasis of brain				
No	3261 (99.2%)	2276 (99%)	985 (99.7%)	0.03
Yes	27 (0.8%)	24 (1%)	3 (0.3%)	
Metastasis of liver				
No	3233 (98.3%)	2263 (98.4%)	970 (98.2%)	0.66
Yes	55 (1.7%)	37 (1.6%)	18 (1.8%)	
Metastasis of lung				
No	2963 (90.1%)	2079 (90.4%)	884 (89.5%)	0.42
Yes	325 (9.9%)	221 (9.6%)	104 (10.5%)	
Tumor size(cm)				
0-4	1474 (44.8%)	1017 (44.2%)	457 (46.3%)	0.28
>4	1814 (55.2%)	1283 (55.8%)	531 (53.7%)	
Tumour number				
1	3176 (96.6%)	2221 (96.6%)	955 (96.7%)	0.89
>1	112 (3.4%)	79 (3.4%)	33 (3.3%)	
Year of diagnosis				
2010	519(15.8%)	365(15.8%)	154(15.6%)	0.95
2011	501(15.2%)	354(15.4%)	147(15.0%)	
2012	520(15.8%)	364(15.8%)	156(15.7%)	
2013	562(17.2%)	390(17.0%)	172(17.4%)	
2014	613(18.6%)	435(18.9%)	178(18.0%)	
2015	573(17.4%)	392(17.1%)	181(18.3%)	

a Marital status, The marital status of patients at diagnosis; SDW, Separated, Divorced and Widowed.

b Testis, NOS: The primary tumor site of the testis was not otherwise specified.

### 3.2 Cox risk regression

**Table 2** demonstrates the results of univariate and multivariate analysis. In univariate Cox regression analysis, significant differences existed in 13 variables (age, marital status, histology, SEER stage, AJCC stage, radiotherapy, chemotherapy, bone metastases, brain metastases, liver metastases, lung metastases, tumor size, and tumor number) (P<0.05). With increased age, HR values also increased, the same as multivariate analysis. Multivariate regression analysis revealed that age, marital status, histologic type, AJCC stage, radiotherapy, chemotherapy, liver metastases, tumor size, and number were independent prognostic factors for OS in TGCT-LVI patients, which were subsequently included in the nomogram construction.

**Table 2:** Univariate and multivariate Cox analyses of the overall survival in patients with TGCT-LVI.

Characteristic	Univariate analysis		P value	Multivariate analysis		P value
	HR	95%CI		HR	95%CI	
Age(year)						
<25	Reference			Reference		
25-44	1.31	0.82-2.09	0.25	1.93	1.18-3.15	<0.01
45-64	2.60	1.57-4.30	<0.001	4.29	2.39-7.71	<0.001
>64	7.74	3.46-17.32	<0.001	12.26	5.04-29.84	<0.001
Race						
Black	Reference			Reference		
White	0.60	0.28-1.28	0.18	0.77	0.35-1.67	0.50
Other	0.97	0.39-2.44	0.96	0.88	0.34-2.27	0.79
Marital status ^a^						
Single	Reference			Reference		
Married	0.67	0.47-0.96	<0.05	0.67	0.45-0.99	<0.05
SDW	1.47	0.84-2.59	0.17	0.95	0.52-1.72	0.86
Unknown	0.34	0.11-1.07	0.06	0.40	0.13-1.29	0.12
Tumor primary site						
Undescended testis	Reference			Reference		
Descended testis	1.21	0.30-4.93	0.79	1.30	0.31-5.46	0.72
Testis, NOS ^b^	0.95	0.23-3.85	0.94	1.02	0.24-4.27	0.98
Laterality						
left	Reference			Reference		
Right	1.37	0.99-1.89	0.06	1.29	0.93-1.80	0.13
Histology						
Seminoma	Reference			Reference		
Nonseminoma	1.44	1.04-2.01	<0.05	1.84	1.18-2.89	<0.01
SEER stage						
Localized	Reference			Reference		
Regional	1.68	1.08-2.61	<0.05	1.60	0.84-3.07	0.16
Distant	7.51	5.16-10.92	<0.001	1.80	0.68-4.79	0.24
AJCC stage						
I	Reference			Reference		
II	1.04	0.61-1.79	0.88	0.83	0.39-1.78	0.64
III	6.70	4.76-9.43	<0.001	3.15	1.32-7.50	<0.01
Radiation						
No	Reference			Reference		
Yes	1.60	1.05-2.42	<0.05	1.69	1.02-2.78	<0.05
Chemotherapy						
No	Reference			Reference		
Yes	1.53	1.09-2.13	<0.05	0.59	0.36-0.97	<0.05
Metastasis of bone						
No	Reference			Reference		
Yes	8.34	3.69-18.89	<0.001	1.06	0.43-2.65	0.89
Metastasis of brain						
No	Reference			Reference		
Yes	13.93	7.88-24.62	<0.001	1.94	0.99-3.83	0.06
Metastasis of liver						
No	Reference			Reference		
Yes	11.57	6.97-19.18	<0.001	3.06	1.71-5.47	<0.001
Metastasis of lung						
No	Reference			Reference		
Yes	6.04	4.34-8.41	<0.001	1.16	0.64-2.10	0.63
Tumor size(cm)						
0-4	Reference			Reference		
>4	5.07	3.23-7.96	<0.001	3.23	2.02-5.17	<0.001
Tumor number						
1	Reference			Reference		
>1	2.76	1.59-4.79	<0.001	2.23	1.29-4.07	<0.01

a Marital status, The marital status of patients at diagnosis; SDW, Separated, Divorced and Widowed.

b Testis, NOS: The primary tumor site of the testis was not otherwise specified.

### 3.3 Construction and verification of nomogram

**Figure 2:**
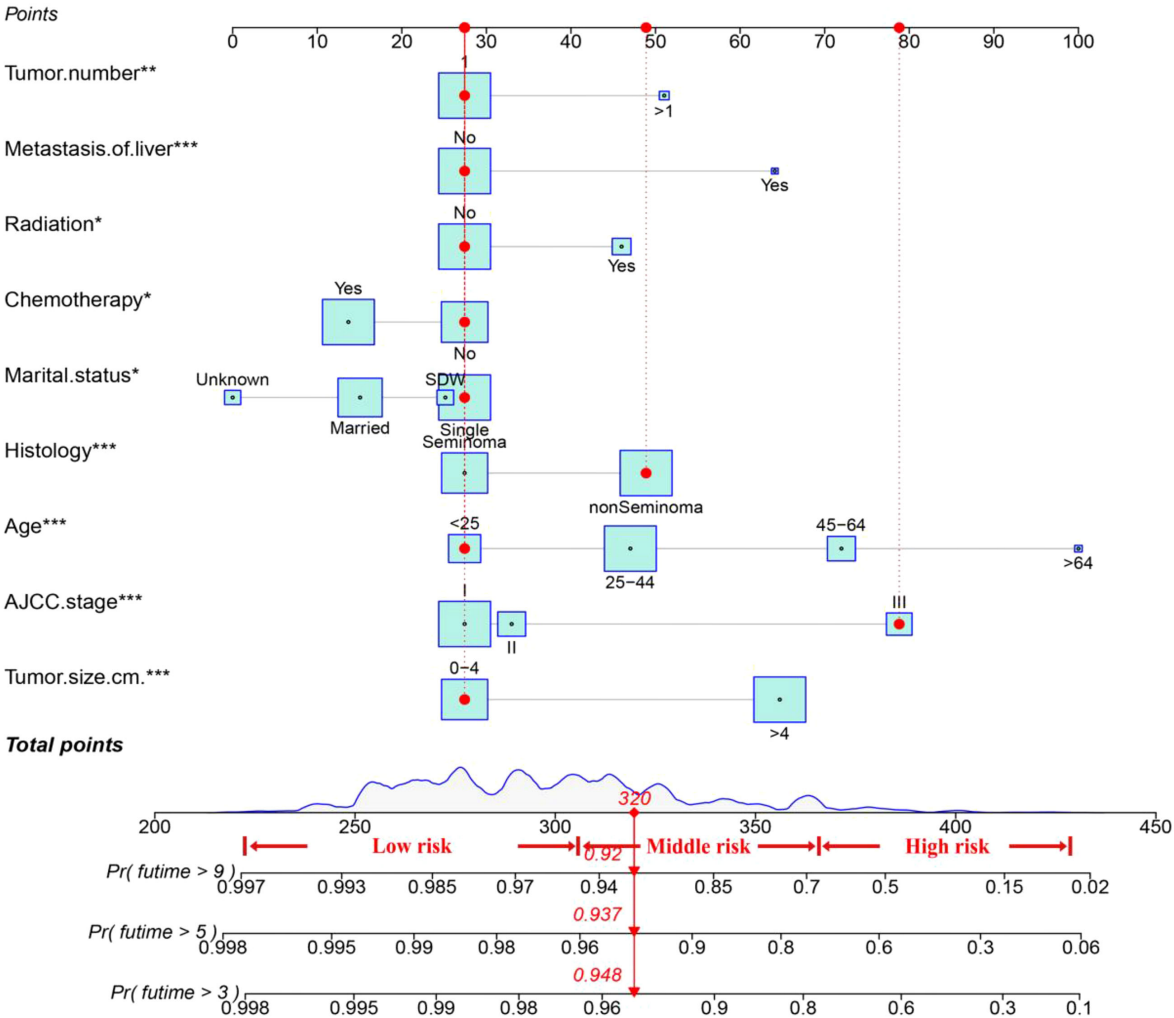
The nomogram for TGCT-LVI patients. An example of predicting the probability of survival for a specific patient by nomogram is shown in the red line. * P < 0.05, ** P < 0.01, *** P < 0.001.

**Figure 2** shows a nomogram constructed by the effective variables and predicting OS at 3, 5, and 9 years in TGCT-LVI patients. An example of predicting the probability of survival for a specific patient by nomogram is shown in red. The total risk score for each patient was calculated using the nomogram, and the total risk score for most patients in this study ranged from 220 to 400.

**Figure 3:**
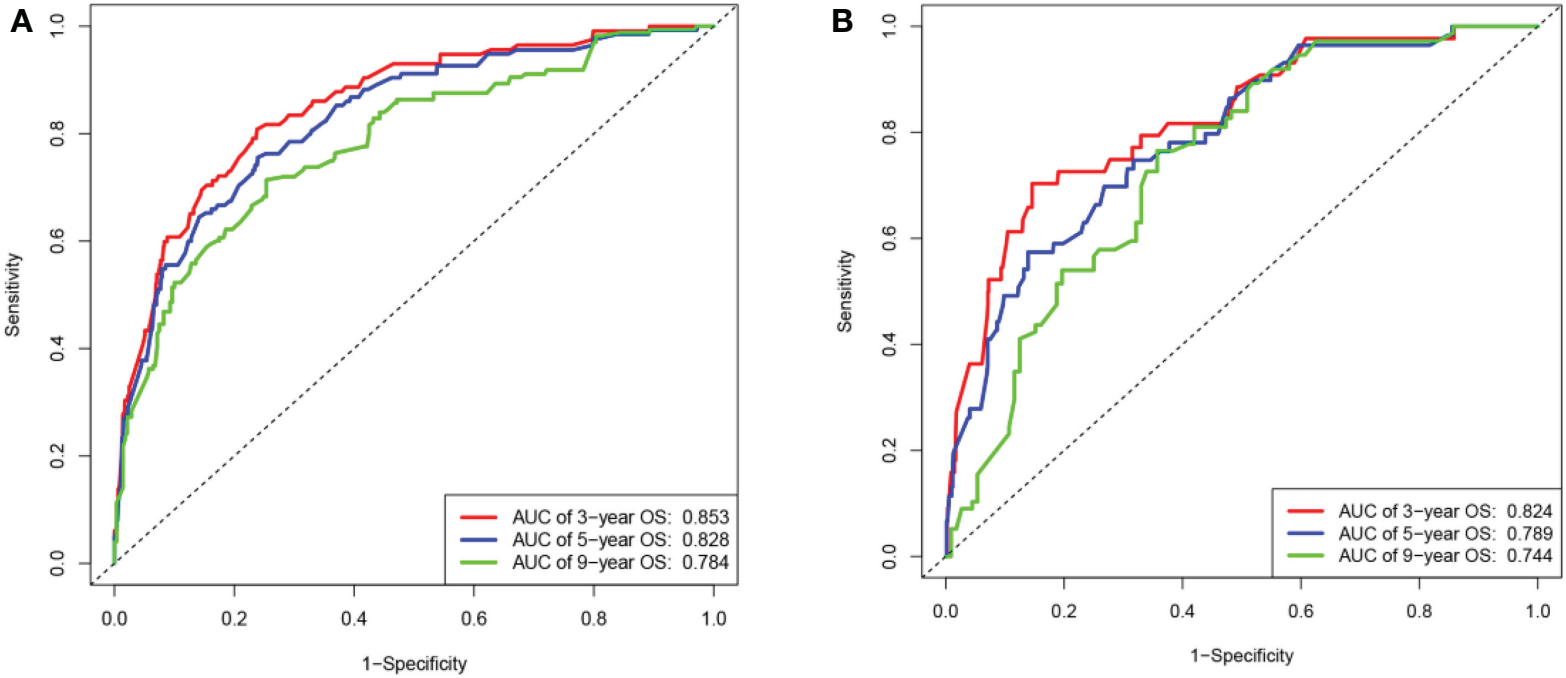
The area under the ROC curves for 3-,5-, and 9-year overall survival predicted by the nomogram. **(A)** Based on the training cohort and **(B)** on the validation cohort.

**Figure 4:**
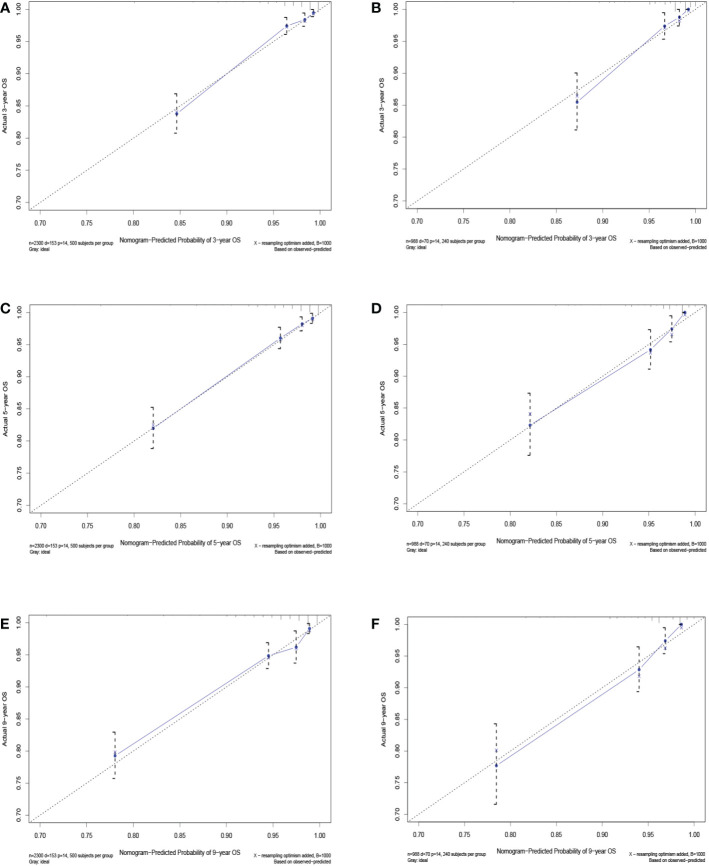
Calibration curves for 3-, 5-, and 9-year overall survival of TGCT-LVI patients in the training cohort **(A, C, E)** and the validation cohort **(B, D, F)**.

The C-index value of the training cohort was 0.821 (95% CI = 0.791–0.851), and the validation cohort was 0.819 (95% CI = 0.786–0.852). The time-dependent ROC curve showed that in the training cohort, the model predicted that the AUC values of 3, 5, and 9 years were >0.75, whereas the corresponding values for the validation cohort were >0.74, indicating that the nomogram had good discrimination ability (**Figure 3**). The calibration curve showed that the predicted survival probabilities of the training and validation cohorts were close to the observed survival probabilities (**Figure 4**). Thus, the nomogram of TGCT with LVI had satisfactory discrimination and calibration power.

### 3.4 Comparison of clinical value of nomogram and AJCC stage

**Figure 5:**
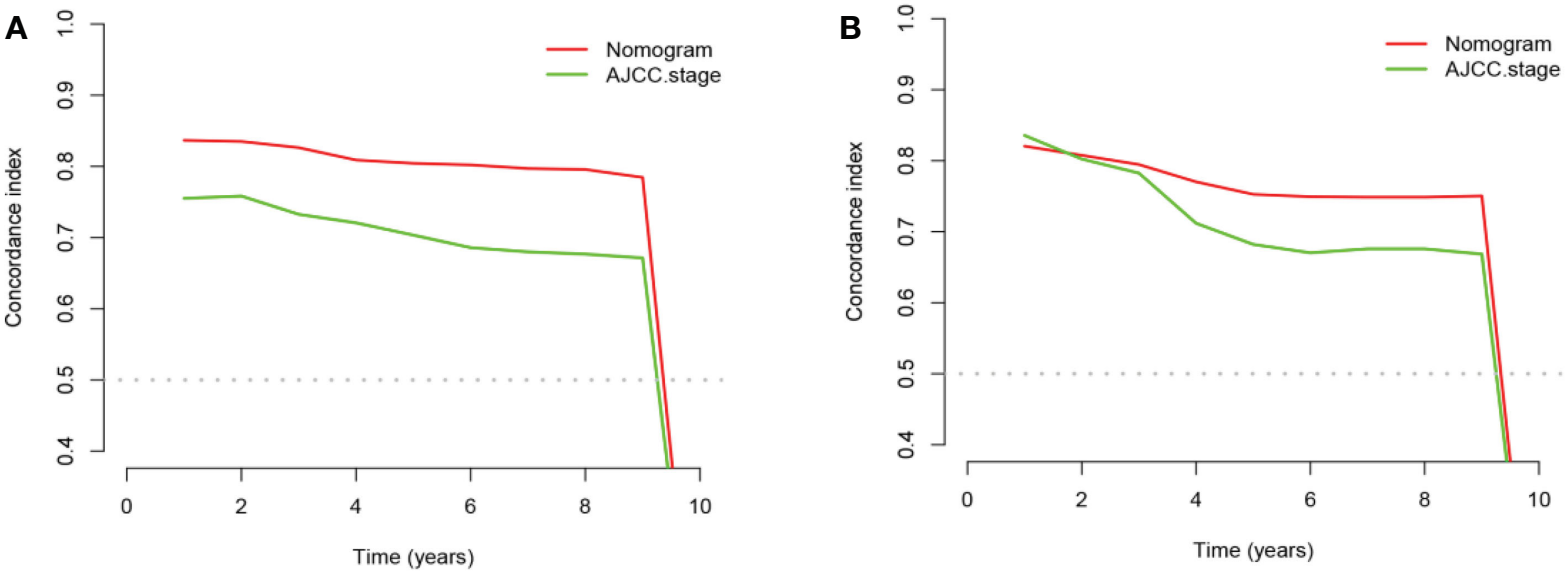
The comparison chart of C-index between nomogram and AJCC stage. **(A)** In the training cohort and **(B)** in the validation cohort.

**Figure 6:**
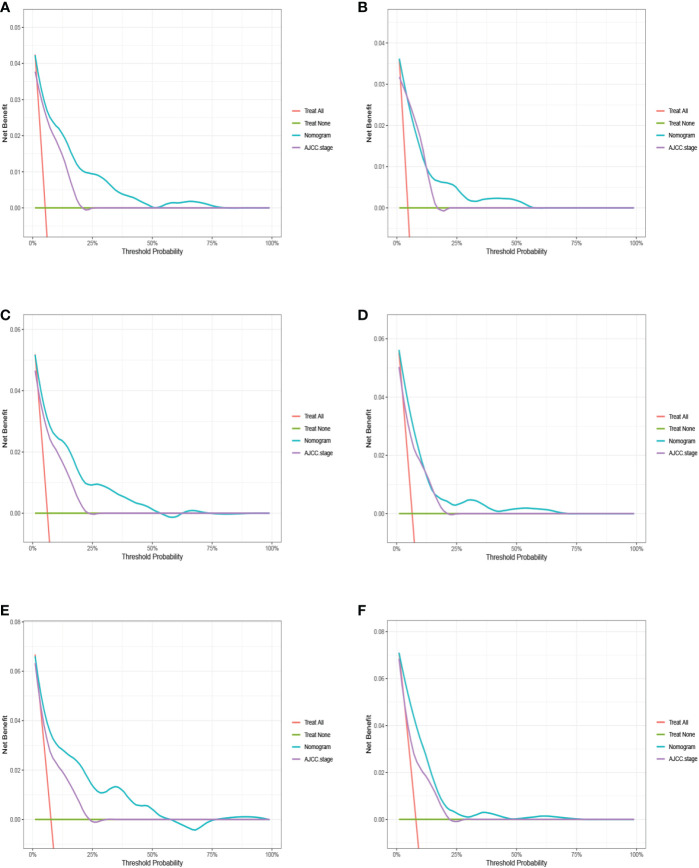
The decision curve analysis (DCA) for 3-, 5-, and 9-year overall survival prediction between nomogram and AJCC stage in the training cohort **(A, C, E)** and validation cohort **(B, D, F)**.

The advantages and disadvantages of the nomogram and AJCC stage were evaluated by analyzing changes in C-index (**Figure 5**), NRI, and IDI. In the training cohort, the C-index associated with the nomogram was significantly higher than the that associated with AJCC stage. The NRI values of 3-, 5-, and 9-year OS were 0.308 (95% CI = 0.182–0.451), 0.274 (95% CI = 0.201–0.440), and 0.295 (95% CI = 0.222–0.489), respectively, whereas the IDI values were 0.093, 0.093, and 0.099 (P<0.01), respectively (**Table 3**). These results suggested that the nomogram predicted prognosis more accurately than did the AJCC stage, and all of these can be verified by the validation cohort. DCA indicated that in both cohorts, the nomogram can better predict 3-, 5-, and 9-year OS and had more net benefits than AJCC stage (**Figure 6**).

**Table 3:** the NRI and IDI values of 3-, 5-, and 9-year OS, with the C-index of nomogram in the training and validation cohorts.

Index	Training cohort			Validation cohort		
	Estimate	95%CI	P value	Estimate	95%CI	P value
NRI						
For 3-year OS	0.308	0.182-0.451		0.294	0.021-0.485	
For 5-year OS	0.274	0.201-0.440		0.138	0.075-0.470	
For 9-year OS	0.295	0.222-0.489		0.220	0.142-0.663	
IDI						
For 3-year OS	0.093	0.063-0.155	<0.01	0.066	0.029-0.172	<0.01
For 5-year OS	0.093	0.066-0.146	<0.01	0.063	0.042-0.160	<0.01
For 9-year OS	0.099	0.067-0.166	<0.01	0.067	0.037-0.157	<0.01
C-index						
The nomogram	0.821	0.791-0.851		0.819	0.786-0.852	

### 3.5 A new risk-stratification system based on nomogram

**Figure 7:**
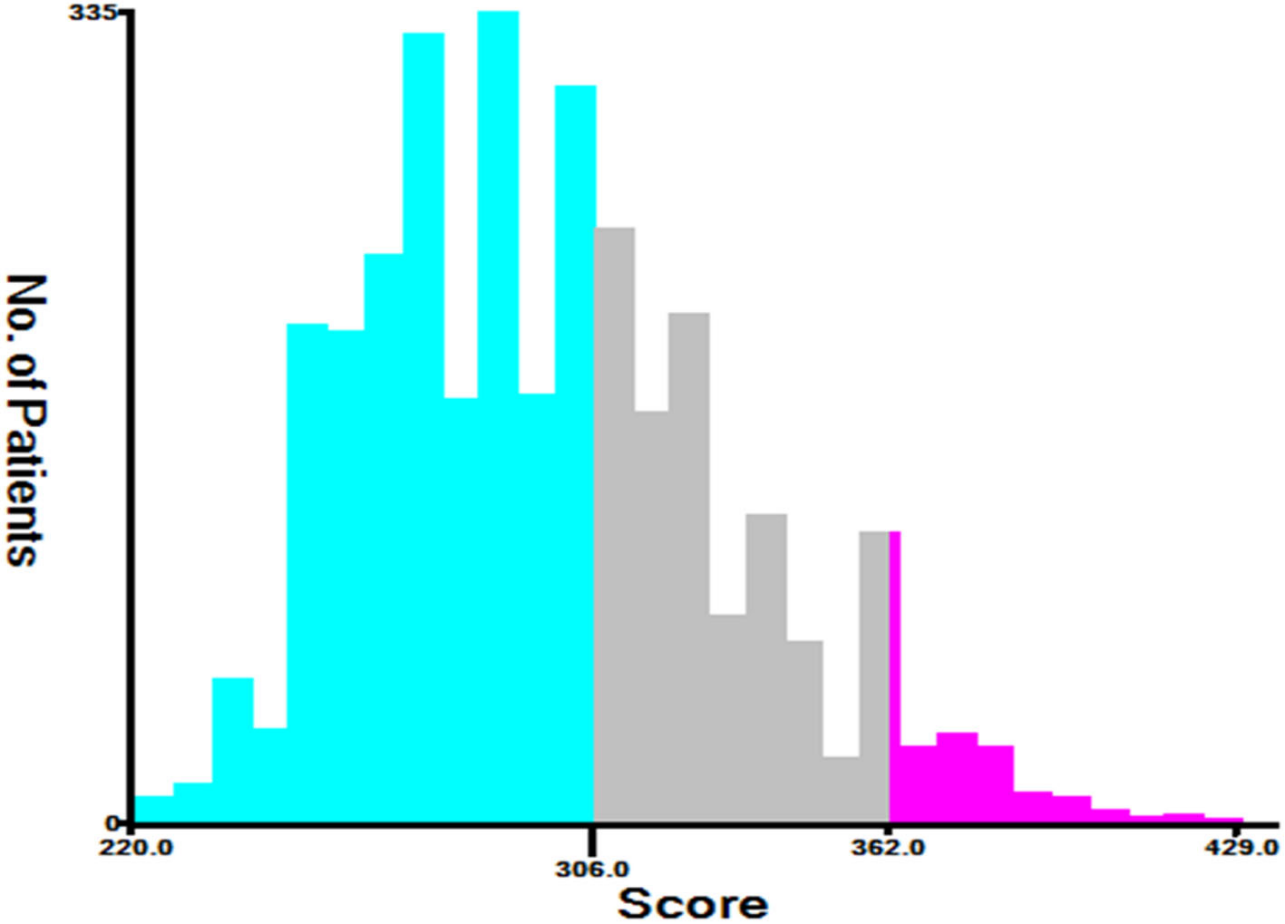
The optimal cut-off for the total scores gained by X-Tile. The total risk scores for most patients in this study ranged from 220 to 400 (low risk: 220≤ total score < 306, medium risk: 306≤ total score < 362, and high risk: 362≤ total score<429).

**Figure 8:**
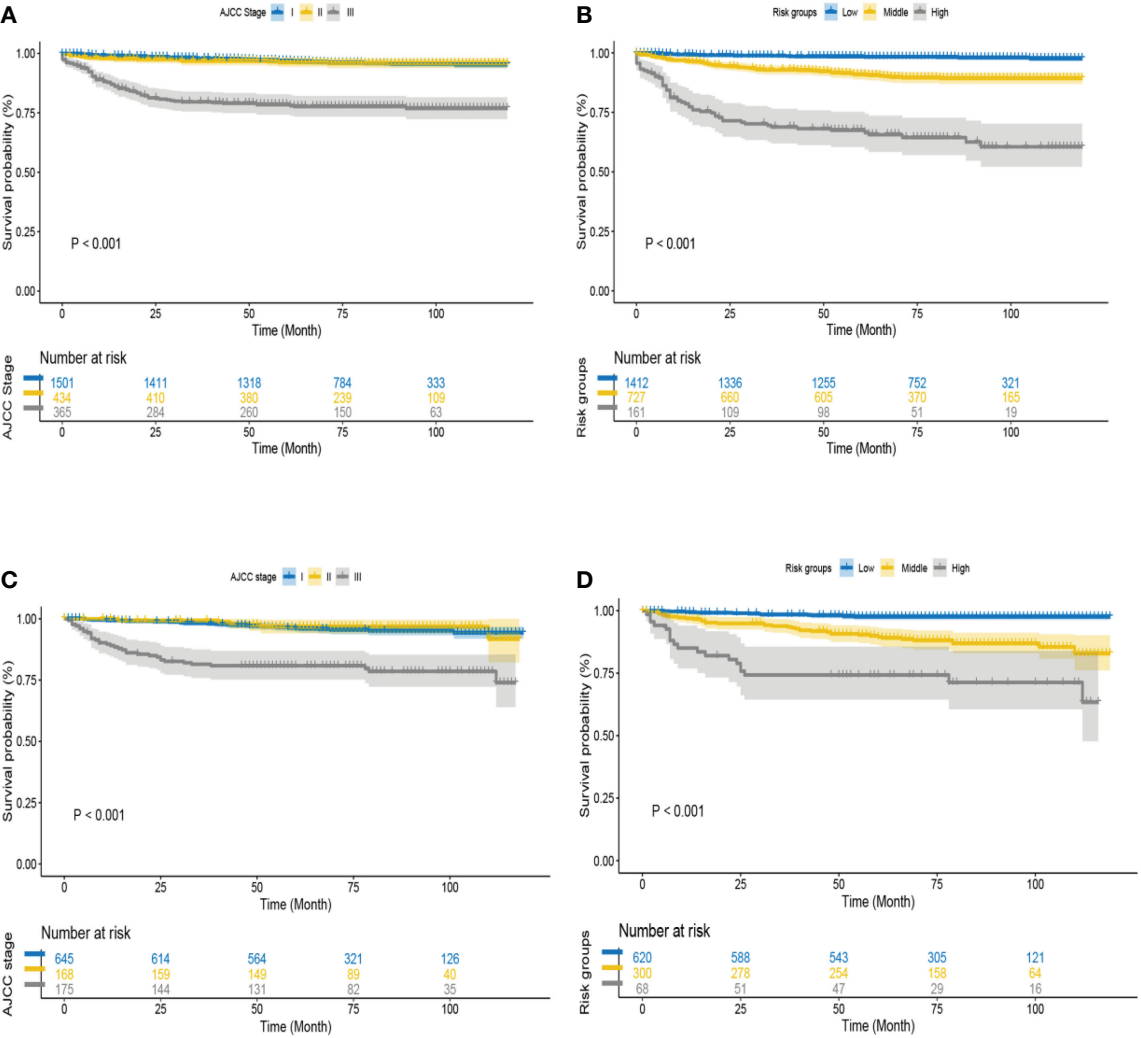
The Kaplan-Meier curve of overall survival for TGCT-LVI patients. **(A)** AJCC staging system and **(B)** a new risk stratification system in the training cohort. **(C)** AJCC staging system and **(D)** a new risk stratification system in the validation cohort.

This study pooled the total scores calculated using the nomogram for all patients. Risk stratification was performed according to the analysis results of X-tile software. All TGCT-LVI patients were divided into three risk groups: low risk (total score < 306), medium risk (306 ≤ total score < 362), and high risk (total score ≥ 362) (**Figure 7**). The Kaplan–Meier curve showed significant differences among the three risk groups, and the new risk-stratification system had better ability to identify patients, whereas the AJCC stage system had limited ability to distinguish between low- and medium-risk patients across all cohorts (**Figure 8**).

## 4 Discussion

Studies have shown that although TGCTs had a high single cure rate, their tendency to relapse increased the likelihood of poor prognosis. Warde et al. found that LVI is an independent and important prognostic factor for tumor recurrence, and the risk of occult metastasis in patients with this factor significantly increases as shown by a pooled analysis of patients with stage I testicular seminoma (14). Subsequently, this factor was shown to apply equally to patients with non-seminomas (20, 21). Prospective studies have revealed that grouping patients with LVI as a high-risk factor can effectively guide postoperative treatment and help reduce the risk of postoperative recurrence and poor prognosis. Accordingly, the present work aimed to construct a nomogram to predict the prognosis of patients with TGCT-LVI.

Some previous studies have also addressed the factors that may affect OS in TGCT patients, such as age, tumor size, tissue type, radiotherapy, and chemotherapy (17, 22). However, these studies do not consider other clinical variables affecting the prognosis of TGCT patients, and LVI is included only as a variable with or without consideration. They also do not note the specificity of TGCT-LVI patients. Our research fully considered these influencing factors and treated TGCT-LVI patients as a separate and special group. Age and histology are known independent risk factors. The median age of diagnosis in patients with seminoma and non-seminoma has been shown to be 37 and 30 years, respectively (9, 21), consistent with our conclusion that tumors tended to occur between 25 and 44 years of age. Compared with seminoma, non-seminoma is more aggressive and likely to induce occult metastasis, resulting in tumor recurrence and poor prognosis. Regarding marital status, we found a beneficial effect of being married on the OS of TGCT-LVI patients, which may be related to multifactorial psychosocial effects at all stages of the disease process, including early detection, treatment adherence, treatment decisions, and adherence to post-treatment monitoring or follow-up (23–26). Meanwhile, we noticed that because the first impression of unmarried patients was a lack of social support, oncologists were more inclined to recommend non-intensive treatment regimens for them (23). As for tumor size and number, Warde (14) and Christian (26) noted that tumors (>4 cm) or multiple tumors often indicate a poor prognosis, which may be linked to increased risk of early metastasis and expanded surgical clearance. Liver metastases are included in most TGCT stage/prognostic factors system and clearly defined as poor (27, 28). Patel’s study of 969 patients diagnosed with stage III TGCT combined with distant metastases has shown that approximately 20% of patients have liver metastases, and the 3-year CSS rate is 64.5% (29). This finding suggests that liver metastases are associated with prognosis in patients with TGCT-LVI. Once diagnosed, radical orchiectomy is the recommended first treatment for patients. However, more evidence is needed regarding the ultimate benefit of significant improvement in patients through surgery combined with adjuvant therapy. Adjuvant radiotherapy is primarily suitable for testicular seminoma tumors that are extremely sensitive. Indeed, studies have found that moderate doses of adjuvant radiotherapy can effectively reduce the rate of tumor recurrence (30), but it has the disadvantage of easily increased risk of secondary malignancies (31). Adjuvant chemotherapy is more common in the treatment of tumors of both histologic types, and patients with TGCT-LVI often receive clinical therapy with a corresponding cycle of BEP regimens under the stage of AJCC testicular tumors (32–34). Compared with radiotherapy, chemotherapy is superior in inhibiting distant metastases and avoiding secondary malignancies *in situ* (35). It can also significantly improve the postoperative prognosis of TGCT-LVI patients by using our nomogram.

The AJCC stage system is an internationally accepted tumor stage system and the basis for treatment decisions and prognosis assessment in TGCT-LVI patients. However, its ability to distinguish between low- and intermediate-risk patients is insufficient in this study. The nomogram has been demonstrated by multiple indicators to outperform traditional AJCC stage in planning clinical decision making and prognosis prediction. By scoring the relevant variables in the nomogram, we constructed a new risk-stratification system for TGCT-LVI patients. The Kaplan–Meier curve showed that the new risk-stratification system was also better than the traditional AJCC stage system in identifying different risk groups. On account of the worst prognosis in high-risk group, the patients of this group should be given more attention or care. They should also be more cautious in selecting the treatment and medication to avoid the toxic/side effects of drugs.

Our study has some limitations. Due to the small sample size of TGCT-LVI patients, we were unable to collect data about cases without radical orchiectomy and with concurrent bilateral TGCTs sufficient for statistical analysis. Second, the SEER database did not publish data on surgical margins, i.e., whether two or more surgeries were performed, radiation dose, chemotherapy cycles, and dose of medication. Consequently, we did not count and evaluate these factors. In addition, because of the small proportion of testicular tumor cases among all male tumor cases, we included OS as an outcome of the study event to increase the number of study cases and the accuracy of prognostic prediction for the model. Further validation of the model results with CSS as the outcome of the study event is still needed in the future. Finally, this work needs to be combined with large-scale, multicenter prospective studies in other countries to verify the clinical application value of the nomograms.

## 5 Conclusion

Based on the SEER database, we summarized the clinical features of TGCT-LVI patients and found that age, marital status, histology, tumor size, tumor number, AJCC stage, adjuvant radiotherapy, chemotherapy, and liver metastases were independent prognostic factors for OS in patients with TGCT-LVI. We also established comprehensive nomograms and a new prognostic risk-stratification system to assess the OS in this group of patients by using these factors. Internal verification showed that the new risk-stratification system was superior to the AJCC stage system. Therefore, it has a good application prospect and can guide the personalized treatment and prognosis of clinical patients.

## Author’s note

**Hu Ke,** E-mail: 1784923593@qq.com

**Shengming Jiang,** E-mail: jackjiangsm@163.com

**Ziqi He,** E-mail: ziqihe1990@163.com

**Yunhe Xiong,** E-mail: xiongyunhe@whu.edu.cn

## Data availability statement

The datasets presented in this study can be found in online repositories. The names of the repository/repositories and accession number(s) can be found in the article/supplementary material.

## Author contributions

HK, SJ and ZH: project development, data analysis, manuscript writing/editing. QS, CD, and DY: data collection/management. JL, XS, JZ: data analysis. CS: formal analysis, writing-review and editing. YX: project development, formal analysis, writing-review and editing. All authors contributed to the article and approved the submitted version.
